# Nanospan, an alternatively spliced isoform of sarcospan, localizes to the sarcoplasmic reticulum in skeletal muscle and is absent in limb girdle muscular dystrophy 2F

**DOI:** 10.1186/s13395-017-0127-9

**Published:** 2017-06-06

**Authors:** Angela K. Peter, Gaynor Miller, Joana Capote, Marino DiFranco, Alhondra Solares-Pérez, Emily L. Wang, Jim Heighway, Ramón M. Coral-Vázquez, Julio Vergara, Rachelle H. Crosbie-Watson

**Affiliations:** 1 Department of Integrative Biology and Physiology, University of California, Los Angeles, 610 Charles E. Young Drive East, Terasaki Life Sciences Building, Los Angeles, CA 90095 USA; 2 Department of Neurology, David Geffen School of Medicine, University of California, Los Angeles, 610 Charles E. Young Drive East, Terasaki Life Sciences Building, Los Angeles, CA 90095 USA; 3 Center for Duchenne Muscular Dystrophy, University of California, Los Angeles, Los Angeles, CA USA; 4 Department of Physiology, David Geffen School of Medicine, University of California, Los Angeles, Los Angeles, CA USA; 5 Sección de Estudios de Posgrado e Investigación, Escuela Superior de Medicina, Instituto Politécnico Nacional, Mexico City, Mexico; 6 Cancer Communications and Consultancy Ltd, Knutsford, Cheshire UK; 7 Molecular Biology Institute, University of California, Los Angeles, Los Angeles, CA USA; 8 Present Address: Biofrontiers Institute, Department of Molecular, Cellular and Developmental Biology, University of Colorado, Boulder, CO USA; 9 Present Address: Department of Oncology and Metabolism, University of Sheffield, Sheffield, UK

**Keywords:** Duchenne muscular dystrophy, Dystrophin, Limb girdle muscular dystrophy, Microspan, Nanospan, Sarcolemma, Sarcoplasmic reticulum, Sarcospan, Transverse tubule

## Abstract

**Background:**

Sarcospan (SSPN) is a transmembrane protein that interacts with the sarcoglycans (SGs) to form a tight subcomplex within the dystrophin-glycoprotein complex that spans the sarcolemma and interacts with laminin in the extracellular matrix. Overexpression of SSPN ameliorates Duchenne muscular dystrophy in murine models.

**Methods:**

Standard cloning approaches were used to identify nanospan, and nanospan-specific polyclonal antibodies were generated and validated. Biochemical isolation of skeletal muscle membranes and two-photon laser scanning microscopy were used to analyze nanospan localization in muscle from multiple murine models. Duchenne muscular dystrophy biopsies were analyzed by immunoblot analysis of protein lysates as well as indirect immunofluorescence analysis of muscle cryosections.

**Results:**

Nanospan is an alternatively spliced isoform of sarcospan. While SSPN has four transmembrane domains and is a core component of the sarcolemmal dystrophin-glycoprotein complex, nanospan is a type II transmembrane protein that does not associate with the dystrophin-glycoprotein complex. We demonstrate that nanospan is enriched in the sarcoplasmic reticulum (SR) fractions and is not present in the T-tubules. SR fractions contain membranes from three distinct structural regions: a region flanking the T-tubules (triadic SR), a SR region across the Z-line (ZSR), and a longitudinal SR region across the M-line (LSR). Analysis of isolated murine muscles reveals that nanospan is mostly associated with the ZSR and triadic SR, and only minimally with the LSR. Furthermore, nanospan is absent from the SR of δ-SG-null (Sgcd^−/−^) skeletal muscle, a murine model for limb girdle muscular dystrophy 2F. Analysis of skeletal muscle biopsies from Duchenne muscular dystrophy patients reveals that nanospan is preferentially expressed in type I (slow) fibers in both control and Duchenne samples. Furthermore, nanospan is significantly reduced in Duchenne biopsies.

**Conclusions:**

Alternative splicing of proteins from the SG-SSPN complex produces δ-SG3, microspan, and nanospan that localize to the ZSR and the triadic SR, where they may play a role in regulating resting calcium levels as supported by previous studies (Estrada et al., Biochem Biophys Res Commun 340:865–71, 2006). Thus, alternative splicing of SSPN mRNA generates three protein isoforms (SSPN, microspan, and nanospan) that differ in the number of transmembrane domains affecting subcellular membrane association into distinct protein complexes.

**Electronic supplementary material:**

The online version of this article (doi:10.1186/s13395-017-0127-9) contains supplementary material, which is available to authorized users.

## Background

Integral and peripheral membrane proteins of the dystrophin-glycoprotein complex, or DGC, function to provide a physical linkage across the muscle membrane to connect the extracellular matrix with the F-actin cytoskeleton [1, 2]. The core components of the DGC include dystrophin, sarcoglycans (SGs), sarcospan (SSPN), dystroglycan (α-, β-DG), and syntrophins [3–7]. The SG subcomplex of the DGC is comprised of four single-pass transmembrane glycoproteins, referred to as α-, β-, γ-, and δ-SG [8]. SSPN, a 25-kDa integral membrane protein with structural homology to the CD20 superfamily of proteins that also exhibits some similarities with the tetraspanins [1], interacts with the SGs at the sarcolemma to form a tight subcomplex (SG-SSPN) that anchors α-DG to the cell surface and contributes to the maintenance of cell integrity [9]. Autosomal recessive limb girdle muscular dystrophy (AR-LGMD) types 2D, 2E, 2C, and 2F are caused by primary mutations in α-, β-, γ-, and δ-SG genes, respectively. Lack of expression of any one of the SGs results in specific loss of the entire SG-SSPN subcomplex, destabilization of α-DG, and sarcolemma damage [8–10].

While the loss of muscle cell adhesion complexes that render the sarcolemma susceptible to contraction is widely accepted as the initial insult to dystrophic muscle, there is strong support for dysfunctional calcium handling in muscle fibers that contributes to disease progression. Electrophysiological studies have revealed impaired excitation-contraction coupling in dystrophin-deficient (*mdx*) muscle, including decreased calcium release from the sarcoplasmic reticulum (SR) in response to voltage activation in *mdx* myofibers [11–14]. Structural and functional defects in the SR calcium release channel ryanodine receptor 1 (RyR1) have contributed to the “leaky” channel model. Elegant studies from Marks and colleagues [15–17] have revealed an increase in S-nitrosylation of cysteine residues of RyR1 in *mdx* muscle leading to disruption of RyR1 macrocomplexes (including depletion of calstabin1) that regulates calcium release from the SR [15–17]. Defective mechanotransduction signaling caused by reactive oxygen species (ROS) contributes to *mdx* calcium dysfunction in a mechanism that includes increased sarcolemma calcium influx through mechanosensitive calcium channels [18–20]. The relevance of SR function to disease progression in the dystrophinopathies and sarcoglycanopathies is becoming clear as SERCA1 overexpression in *mdx* and δ-SG-null (Sgcd^−/−^) mice reduces central nucleation, tissue fibrosis, and membrane damage [21]. Furthermore, introduction of SERCA1 reversed SR calcium reuptake defects and reduced total cytosolic calcium that characterizes dystrophic muscle [21]. These studies are bolstered by additional reports that introducing SERCA2 or a dominant-negative Orai1 reduces dystrophic pathology by ameliorating SR calcium handling defects [21, 22].

Using confocal and electron microscopy, Ueda and colleagues demonstrated that γ- and δ-SG are present in the SR membranes in addition to their localization with dystrophin at the sarcolemma while α- and β-SG are confined to the sarcolemma [23]. This finding suggested that γ- and δ-SG must have additional functions in the SR membranes independent of their role in the DGC at the sarcolemma. Moreover, it was later reported that an alternatively spliced isoform of δ-SG called δ-SG3, in which the C-terminus is replaced by 10 amino acids, is also exclusively expressed at the SR membranes of skeletal muscle [24]. Based on the location of this protein and the abnormalities in calcium homeostasis across the SR membranes previously described in δ-SG-deficient animals, the authors hypothesize that δ-SG3 could be involved in the regulation of appropriate localization and/or maintenance of the proteins participating in calcium homeostasis in the SR.

We have previously identified an alternatively spliced isoform of SSPN that is not localized to the sarcolemma [25]. SSPN messenger RNA (mRNA) undergoes alternative splicing to generate a novel smaller protein that we named microspan (μSPN) [25], which is enriched in both the light and heavy fractions of the SR membrane (LSR and HSR, respectively) [26]. Analysis of the SR fractions revealed that while γ-SG, δ-SG, δ-SG3, and μSPN are all present in the LSR, only δ-SG3 and μSPN seem to be expressed in the HSR. Furthermore, evaluation of the expression of these proteins in skeletal muscle of Sgcd^−/−^ mice showed that loss of δ-SG expression results in secondary loss of γ-SG and μSPN. The findings raise the possibility that these proteins may be associated as a complex in the SR membranes and that δ-SG (or δ-SG3) may play a role in the stabilization and/or localization of γ-SG and μSPN expression at the SR [26]. Assessment of the Ca^2+^ release and uptake of the LSR and HSR from wild-type and Sgcd^−/−^ mice showed that, whereas the Ca^2+^ release from HSR vesicles from these mice is not different, Ca^2+^ efflux (mainly through SERCA1) is considerably increased in LSR vesicles from Sgcd^−/−^ mice [26]. In addition, the authors showed modifications in the thermal stability and enthalpy of SERCA1 in LSR vesicles from Sgcd^−/−^ mice, suggesting that absence of the LSR-specific SG-SSPN complex (γ-SG, δ-SG, δ-SG3, and μSPN) affects the structure and function of SERCA1 [26].

In the current study, we report the identification of a third protein generated from alternate splicing of SSPN mRNA. We designate it “nanospan” or nSPN as the predicted molecular weight (10.5 kDa) is smaller than that of either SSPN or μSPN. We demonstrate that nSPN is localized to the SR membranes and is reduced in the dystrophic disease context.

## Methods

### cDNA library screen

**Fig. 1:**
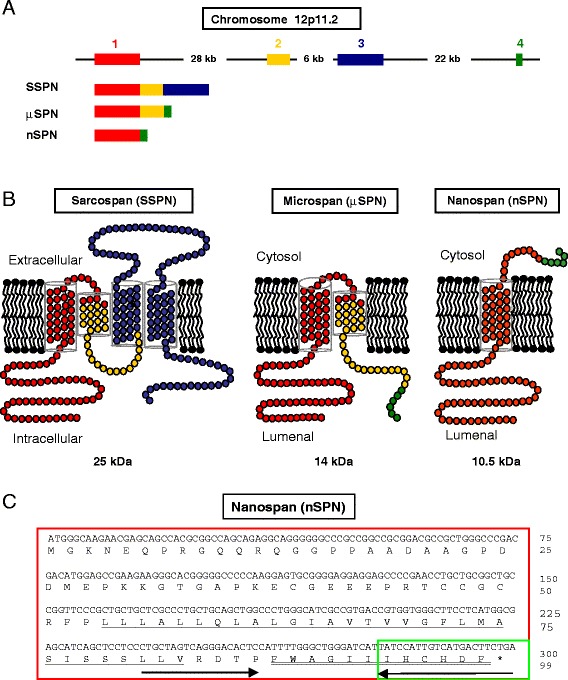
Alternative splicing of SSPN generates μSPN and nSPN. **a** A schematic representation of the genomic organization of the *SSPN* gene on human chromosome 12p11.2. Exons are depicted as *solid boxes* and are color-coded. Exon 1 (*red*), exon 2 (*yellow*), exon 3 (*blue*), exon 4 (*green*), and the introns (*black lines*) are depicted. The approximate size of each intron is indicated (kb). The mRNA splicing patterns generating SSPN, μSPN, and nSPN are also shown. **b** Predicted membrane topologies for SSPN, μSPN, and nSPN are illustrated in three separate schematic diagrams. Each polypeptide region is color-coded according to its corresponding exon. SSPN possesses four transmembrane domains (TM1 to TM4), a small extracellular loop (SEL), and a large extracellular loop (LEL). μSPN contains two transmembrane domains (TM1 and TM2). N- and C-termini of μSPN are strongly predicted to be intracellular using topology algorithms. nSPN retains the first transmembrane domain of SSPN (TM1) and is predicted to have an extracellular C-terminus. The predicted molecular weight of each SSPN-related protein is indicated. **c** The primary and deduced amino acid sequence of human nSPN is shown in single-letter code. Each exon and its deduced amino acid sequence are color-coded as follows: exon 1 (*red*) and exon 4 (*green*). The open reading frame of nSPN encodes a polypeptide of 99 amino acids. The predicted transmembrane domain (TM1) is *underlined*. The 12-amino-acid region used as an antigen for polyclonal antibody (Rabbit 20) production is *double-underlined. Arrows* indicate forward and reverse primers, summarized in Table 1, used in RT-PCR analysis (Additional file 1: Figure S1). The *asterisk* represents the predicted stop codon

A human skeletal muscle complementary DNA (cDNA) library (BD Biosciences Clontech) was screened with probes representing SSPN exons 1 and 3, as described previously [25]. Clones containing exon 1, but not exon 3, were purified and PCR-amplified using primers in the vector arms. PCR products were cloned into pCR^@^2.1-TOPO^@^ (Invitrogen, Carlsbad, CA) and subjected to direct DNA sequencing (UCLA Sequencing and Genotyping Core Facility). Clones containing exon 1 spliced to a novel exon, named exon 4, were identified. An open reading frame was identified and predicted to encode a small, 10.5-kDa protein that was named nanospan (abbreviated as nSPN), as depicted (Fig. 1).

As a complementary approach, the nSPN cDNA was amplified by reverse transcriptase polymerase chain reaction (RT-PCR) from 2.5 μg of Marathon-Ready™ human skeletal muscle cDNA (BD Biosciences Clontech, San Jose, CA) using forward primers in exon 1 (SSPNATGF: 5′-ATGGGCAAGAACGAGCAG-3′; GenBank AF016028; human SSPN) and reverse primers in the 3′ untranslated region (UTR) of μSPN located 26 nucleotides (nt) downstream of the stop codon (Ex3vReverse: 5′-TCCATTCCAAATGGCTTGAT-3′). Table 1 provides a complete list of PCR primers used in the study. A second round of PCR was performed as above using the exon 1 forward primer (SSPNATGF) and a second, nested reverse primer located between the boundary of exon 4 with 2 nt in exon 1 (Ex1/4R: 5′-TCAGAAGTCATGACAATGGATAA-3′; GenBank EU433933; human μSPN). PCR was performed with an Eppendorf gradient thermocycler (Westbury, NY) using the following conditions: 35 cycles of denaturation at 94 °C for 15 s, annealing at 52 °C for 90 s, and extension at 72 °C for 2 min, followed by 72 °C for 10 min. PCR products were subjected to agarose gel electrophoresis and gel extraction with QIAEX II Gel Extraction System (Qiagen, Valencia, CA). Two PCR products were isolated, cloned into pCR2.1-TOPO^@^ and subjected to direct DNA sequencing (Additional file 1 : Figure S1). One of the fragments (387 base pairs (bp)) encoded μSPN (NCBI EU433933), which served as a positive control for the PCR. A second, smaller fragment (300 bp) represented direct splicing of exon 1 to exon 4 to create a complete open reading frame (ORF) with an initiator methionine and a stop codon (Fig. 1b). The ORF is predicted to encode nSPN (Fig. 1c). The NCBI/GenBank accession number for the human nSPN sequence is DQ897666.

**Table 1:** RT-PCR primers for SSPN, μSPN, and nSPN amplification

Primer name	Sequence (5′ ➔ 3′)	NCBI accession no.	Usage	Location within SSPN	Cross-reactivity	Species
SSPNATGF	ATGGGCAAGAACGAGCAG	AF016028	Forward primer, 1st and 2nd PCR	Exon 1	SSPN, μSPN, and nSPN	Human
Ex3vReverse	TCCATTCCAAATGGCTTGAT	EU433933	Reverse primer, 1st PCR	3′ UTR (26 nt downstream of stop codon)	μSPN, nSPN	Human
Ex1/4R	TCAGAAGTCATGACAATGGATAA	EU433933	Reverse primer, 2nd PCR; probe, Northern blot	Exon 1 and exon 4 boundary	μSPN, nSPN	Human
SSPN118F	CTGCTAGTCAGGGACACTC	AF016028	Forward primer, RT-PCR	Exon 1	SSPN, μSPN, and nSPN	Human
Ex4R	TTGATTCTGAGAACATTCTCTTTC	EU433933	Reverse primer, RT-PCR	3′ UTR (7 nt from stop codon)	μSPN, nSPN	Human

### RT-PCR multiple tissue analysis

Total RNA (1 μg) from human tissues was used to synthesize cDNA using the Promega Reverse Transcription System (Promega, Madison, WI). RT-PCR was performed using a forward primer in exon 1 (SSPN1118F: 5′-CTGCTAGTCAGGGACACTC-3′; GenBank AF016028; human SSPN) and a reverse primer in the 3′ UTR of nSPN that is 7 nt downstream of the stop codon (Ex4R: 5′-TTGATTCTGAGAACATTCTCTTTC-3′; GenBank EU433933; human μSPN). Please refer to Table 1 for supporting oligonucleotide information. RT-PCR conditions were performed as previously described [25, 27].

### Northern blotting

Adult human multiple tissue Northern blots (Clontech Inc.) containing 2 μg of poly-A^+^ RNA per lane were probed with oligonucleotides spanning exon 1 and exon 4 as described previously [1] and in Table 1.

### Generation of nSPN antibodies

Polyclonal nSPN antibodies were produced by injecting 500 μg of a synthetic peptide (Peptide 20 CAARRFWAGIIIHCHDF, New England Peptide, Gardner, MA) coupled to KLH into a New Zealand white rabbit (Rabbit 20, Charles River, Wilmington, MA), using previously described methods [1, 25]. The peptide sequence spans the junction between exons 1 and 4 (Fig. 1a, double underline), which is unique to nSPN. Antibodies were affinity-purified from rabbit serum using nSPN peptides coupled to BSA and are referred to as R20. Antibodies to the N-terminal region of SSPN (SSPN amino acid 1–26; GenBank accession number U02487, Rabbit 3) were previously generated [25]. These antibodies recognize SSPN, μSPN, and nSPN as these isoforms all contain the N-terminal region encoded by exon 1 (Table 2).

**Table 2:** Rabbit polyclonal antibodies to SSPN, μSPN, and nSPN

Antibody (Rabbit #)	Antigen	Location (amino acid region)	Species cross-reactivity	Protein cross-reactivity
3	GST-mouse N-terminal fusion protein	1–26	Mouse	SSPN, μSPN, and nSPN
12 and 13	Human C-terminal peptide	117–128	Human, mouse, rabbit	μSPN
14	Rabbit N-terminal peptide	4–20	Rabbit	SSPN, μSPN, and nSPN
15	Human N-terminal peptide	4–26	Human	SSPN, μSPN, and nSPN
18	Mouse MBP C-terminal fusion protein	186–216	Mouse	SSPN
20	Human C-terminal peptide	88–99	Human, mouse, rabbit	nSPN

### Animal models

*mdx* (C57-BL/10ScSn-*Dmd* ^*mdx*^/J), wild-type (C57-BL/6J), and δ-SG-null (Sgcd^−/−^, B6.129-Sgcd^tm1Mcn^/J) mice were purchased from Jackson Laboratory (Bar Harbor, ME). SSPN-deficient mice [28] were a gift from Kevin P. Campbell, Ph.D. (University of Iowa College of Medicine, Howard Hughes Medical Institute, Iowa City, IA) and are available from Jackson Laboratory. Genotyping was confirmed through established PCRs as described previously [29]. Mice were maintained in the Terasaki Life Sciences Vivarium, and all procedures were performed in accordance with guidelines set by the University of California, Los Angeles Institutional Animal Care and Use Committee (A3196-01).

### Control and DMD protein preparations

Total skeletal muscle lysates were prepared in modified RIPA lysis buffer (1% Nonidet P-40, 0.5% sodium deoxycholate, 0.1% SDS, 1 mM EDTA, 5 mM *N*-ethylmaleimide, 50 mM sodium fluoride, 2 mM β-glycerophosphate, 1 mM sodium orthovanadate, 100 nM okadaic acid, 5 nM microcystin LR, and 20 mM Tris-HCl; pH 7.6). Prior to homogenization, protease inhibitors (0.6 μg/mL pepstatin A, 0.5 μg/mL aprotinin, 0.5 μg/mL leupeptin, 0.75 mM benzamidine, and 0.1 mM PMSF) were added to the lysis buffer. Samples were rotated at 4 °C for 1 h and clarified by centrifugation at 15,000 × *g* for 15 min. Before analysis by SDS-polyacrylamide gel electrophoresis, protein concentrations were analyzed using the DC Protein Assay® (Bio-Rad, Hercules, CA). Clarified lysates (60 μg) were loaded onto 15% SDS-polyacrylamide gels and transferred to nitrocellulose (Millipore Corp., Billerica, MA). Nitrocellulose membranes were subsequently incubated with affinity-purified, polyclonal antibodies to nSPN (Rabbit 20, bleed 2; 1:100) and SSPN (Rabbit 15, bleed 4; 1:100). Horseradish peroxidase-conjugated anti-rabbit IgG (Amersham Pharmacia Biotech, Piscataway, NJ) secondary antibodies were used at a 1:3000 dilution. Coomassie Plus Protein Assay Reagent (Pierce, Rockford, IL, USA) was used to confirm equal loading of control and Duchenne muscular dystrophy (DMD) tissue lysates.

### Immunofluorescence analysis

Transverse (8 μm) sections of control and DMD tissue were prepared utilizing a Leica CM3050 S cryostat (Leica Microsystems, Bannockburn, IL). Sections were blocked with 3% BSA in PBS for 1 h at room temperature (RT). To confirm genotype of human samples, dystrophin C-terminal antibodies (VP-D505, Vector Laboratories Inc.) were used. Dystrophin was detected at the sarcolemma in control tissue, but was absent in DMD biopsies. For co-localization experiments, sections were incubated with antibodies to nSPN (Rabbit 20, bleed 4; 1:10) and slow myosin heavy chain (VP-M667, Vector Laboratories Inc.; 1:50) or fast myosin heavy chain (VP-M665, Vector Laboratories Inc.; 1:25), at 4 °C for 12 h. After washing, sections were incubated with FITC anti-rabbit (Vector Laboratories Inc.) and Cy3 anti-mouse (Jackson ImmunoResearch, West Grove, PA) secondary antibodies diluted 1:500 for 1 h at RT. Sections were washed with PBS and mounted using VECTASHIELD (Vector Laboratories Inc.). Sections were incubated with secondary antibodies alone as a negative control (data not shown). All sections were photographed under identical conditions using an Axioplan 2 fluorescence microscope and AxioVision 3.0 software (Carl Zeiss Inc, Thornwood, NY).

### Isolation of transverse tubular and sarcoplasmic reticulum membranes

Transverse tubules (T-tubules) and SR membranes were obtained from total skeletal muscles dissected from 4-month-old wild-type (WT) and Sgcd^−/−^ mice. Isolation was performed by differential centrifugation and a discontinuous sucrose gradient as previously described [30]. Membrane isolation was performed in the absence of any reducing agent in the buffer medium. The microsomal fraction was placed in a first sucrose gradient of 25, 27.5, and 35% (w/v). The 25/27.5% interface showed the maximal signal for DHPR as determined by immunoblotting, indicating that the microsomal fraction contains T-tubule membranes (not shown). An additional centrifugation through a 25/45% discontinuous sucrose gradient was done to remove Ca^2+^-oxalate-loaded vesicles [31]. Heavy SR and light SR were isolated from the 35% band obtained from the first discontinuous sucrose gradient essentially as previously described [32]. Light SR was detected by the maximum ATPase activity stimulated by Ca^2+^, and HSR was detected by the maximal immunoreactivity with the antibody anti-RyR. Anti-nSPN antibodies were used for immunoblot analysis (R20, B2). Membrane preparations were validated by immunoblot analysis with antibodies to DHPR, RyR, SERCA1, and triadin (data not shown) as described previously [30, 31]. Protein concentration was determined using Coomassie Plus Protein Assay Reagent (Pierce, Rockford, IL, USA) with BSA as the standard.

### Mouse muscle immunohistochemistry

Flexor digitorum brevis and interosseous muscles were dissected from C57-BL/6J and SSPN-null mice. Muscles were slightly stretched and pinned down into a Sylgard-bottomed petri dish containing Tyrode’s solution (145 mM NaCl, 5 mM KCl, 2 mM CaCl_2_, 1 mM MgCl_2_, 10 mM Na-MOPS, and 10 mM dextrose; pH 7.2). Surrounding connective tissue was mechanically removed under a dissecting microscope. Subsequently, muscles were fixed in 2% paraformaldehyde (in Tyrode’s) for 1.5 h at RT. After three washes with PBS (10 min each), muscles were permeabilized (0.2% Triton X-100, 1% FBS in PBS) for 1.5 h at RT, and then washed three times with a 1% FBS solution in PBS (10 min each). When primary antibodies raised in mice were utilized, an extra blocking step was added to the protocol at this point, by employing a Mouse on Mouse (M.O.M.™) basic kit (Vector Laboratories Inc.), following the manufacturer’s instructions. Muscles were then incubated with the primary antibody (diluted in either M.O.M. diluent or 1% FBS in PBS) for 6 h at RT or overnight at 4 °C. After three washes (1% FBS in PBS, 10 min each), muscles were later incubated in the correspondent secondary antibody, labeled with either Alexa 488 or Texas Red (diluted 1:50 either in M.O.M. diluent or 1% FBS in PBS) for 6 h at RT or overnight at 4 °C. Samples were finally washed three times with PBS (10 min each) and then maintained in PBS until imaged. Sequential, not simultaneous, labeling was used when two different antibodies were used.

### Two-photon laser scanning microscopy

Antibody-labeled muscles were pinned down on Sylgard-bottomed petri dishes containing PBS and placed on the stage of an upright microscope (BX51WI, Olympus, Japan) equipped with a tunable Chameleon Ti/Sapphire laser (Coherent) and a Radiance 2000 Scanning Head (Bio-Rad, UK). Images were acquired with a ×20, 0.95 NA (Olympus XLUMPLANFL) water-immersion objective. Muscles labeled with Alexa 488 alone or Alexa 488 and Texas Red were excited with a laser wavelength of 840 nm. Images were simultaneously acquired using the following filter combinations (band-pass/dichroic/band-pass, in nm): second harmonic generation (SHG) and Alexa 488 emission (410-490//495//500-530), Alexa 488 and Texas Red emission (500-530//550//620-660), and SHG and Texas Red emission (402-446//495//615-645). Images were analyzed using commercial (LaserSharp 2000, Bio-Rad, UK) and/or public-domain image analysis software package (ImageJ).

## Results

### Molecular cloning of nanospan, a splice variant of sarcospan

The *SSPN* gene is located on human chromosome 12p11.2 and consists of four exons separated by large introns [1, 33]. The full-length SSPN protein is encoded by three small exons (exons 1, 2, and 3) specifying a 25-kDa protein with four membrane-spanning domains (TM1–4 from the N- to the C-terminus), a short extracellular loop (SEL), and a large extracellular loop (LEL; Fig. 1a, b) [1]. The N- and C-termini are predicted to be intracellular. We have shown that SSPN mRNA undergoes alternative splicing to generate microspan (μSPN) [25]. Exons 1, 2, and 4 encode μSPN, a 14-kDa protein that is predicted to contain an intracellular N-terminus as well as transmembrane domains 1 and 2 (Fig. 1b; amino acids encoded by exons 1 and 2 are in red and yellow, respectively). Exon 4 introduces six new amino acids that are predicted to be intracellular (Fig. 1b; amino acids encoded by exon 4 are in green). μSPN is not present at the sarcolemma, but is localized within the SR membranes [25].

The presence of multiple SSPN-related transcripts that encode alternately spliced isoforms of SSPN is supported by Northern blot analysis in which many SSPN transcripts are present in skeletal and cardiac muscle [1, 33]. All transcripts hybridized to *SSPN* exon 1 probes, suggesting that exon 1 is common to all SSPN isoforms, while exon 3 probes only hybridized to a single band on Northern blots [1, 33]. In our original identification of μSPN, we screened a human skeletal muscle cDNA library separately with exon 1 and exon 3 probes. We identified many clones that hybridized to exon 1, but not exon 3, suggesting the presence of other SSPN-related transcripts. As previously reported [25], four individual clones were isolated with inserts of approximately 4 kb, and one of these clones contained an SSPN splice variant that we named μSPN. In the current study, we report on the DNA sequencing of a second clone, revealing the presence of an ORF flanked by a short 5′ UTR and a long 3′ UTR. The ORF was composed of exon 1 spliced to exon 4 (GenBank DQ897666; Fig. 1).

As a complementary approach, RT-PCR amplification was performed using human skeletal muscle cDNA as a template. The forward primer was designed to hybridize to exon 1, which is common to both SSPN and μSPN (Fig. 1c). However, the reverse primer sequence hybridized to exon 4, which is unique to μSPN (Fig. 1c). PCR assays performed with primers in exons 1 and 4 are predicted to amplify μSPN as well as any other cDNAs hybridizing to the exon 1 and 4 primers. Only two PCR products were isolated and characterized by direct DNA sequencing (Additional file 1: Figure S1). One of the fragments (387 bp) encoded μSPN (NCBI EU433933), which served as a positive control for the PCR. A second, smaller fragment (300 bp) represented direct splicing of exon 1 to exon 4 to create a complete ORF with an initiator methionine and a stop codon (Fig. 1b). The ORF is predicted to encode a polypeptide of 99 amino acids with a calculated molecular weight of 10.5 kDa (Fig. 1c; NCBI DQ897666). We designate this isoform as “nanospan” (nSPN), in keeping with prior nomenclature reflecting the predicted molecular weight of SSPN isoforms (Fig. 1).

SSPN exhibits homology to the CD20 and tetraspanin superfamilies. Topology algorithms predict the N- and C-termini of SSPN are both intracellular, as illustrated in Fig. 1b [1]. μSPN is encoded by splicing of exons 1, 2, and 4 to produce a protein with only two transmembrane domains (TM1–2) likely forming a hairpin loop structure in the plasma membrane [25]. The primary amino acid sequence deduced from the human cDNA of nSPN predicts a protein with a single membrane-spanning domain and a topology distinct from either SSPN or μSPN (Fig. 1c). Exon 1, common to all three splice variants of SSPN, encodes an intracellular N-terminus and one transmembrane domain (Fig. 1a–c; amino acids encoded by exon 1 are in red). In nSPN, exon 1 is directly spliced to exon 4, which encodes only six amino acids followed by a stop codon (coded in green). Although nSPN and μSPN both contain exon 4, their C-terminal regions are predicted to lie on different faces of the membrane. nSPN does not have homology to any other known proteins found in the GenBank, TIGR, or dbEST databases and does not possess conserved sites for glycosylation. TM3, LEL, and TM4 are unique to SSPN, and previous work from our group suggests the major SG binding domain(s) is within LEL [34]. This is consistent with previous data that SSPN, but not μSPN, is an integral component of the DGC [1, 33, 35].

### Nanospan mRNA is expressed in human skeletal muscle

**Fig. 2:**
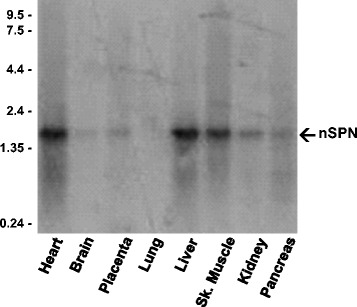
Expression of nSPN mRNA transcript in human tissues. Northern blots containing 2 mg of poly-A^+^ RNA from the indicated human tissues were hybridized with nSPN-specific oligonucleotide. An *arrow* indicates the 1.5-kb nSPN mRNA transcript. Molecular size markers are indicated on the *left* (kb)

To examine the distribution of nSPN mRNA, Northern blot analysis was performed on a broad range of human tissues. Nylon membranes containing poly-A^+^ RNA were probed with oligonucleotides designed to hybridize to nSPN (Table 1), as illustrated in Fig. 1a. We observed an abundant 1.5-kb transcript in heart, liver, and skeletal muscle (Fig. 2). Lower levels of the 1.5-kb nSPN transcript were found in several non-muscle tissues including the brain, placenta, kidney, and pancreas. RT-PCR analysis on cDNA isolated from a variety of human tissues demonstrates nSPN is highly expressed in striated muscles (Additional file 1: Figure S1), supporting Northern blot data.

### Detection of nanospan protein in skeletal muscle

**Fig. 3:**
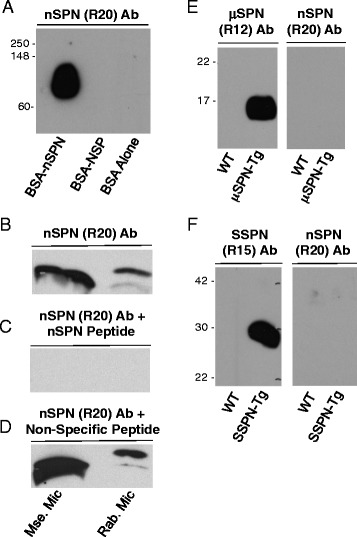
Expression of nSPN protein in skeletal muscle from multiple sources. Immunoblots containing BSA alone, BSA coupled to nSPN (BSA-nSPN), and BSA coupled to nSPN-specific Peptide 20 (BSA-nSPN) (**a**). R20 antibodies recognized an ~11-kDa protein band on immunoblots of total microsomes prepared from mouse and rabbit skeletal muscles (**b**–**d**). Binding of R20 antibodies (R20 Ab) to nSPN was inhibited by nSPN peptides (**c**), but not by non-specific control peptides (**d**). Nitrocellulose membranes of protein lysates from wild-type (non-transgenic) and μSPN-transgenic (Tg) skeletal muscle were probed with μSPN-specific antibodies (R12), which demonstrate cross-reactivity to μSPN-transgenic protein as expected (**e**). However, nSPN antibodies do not cross-react with μSPN (**e**). Total protein lysates from wild-type (non-transgenic) and SSPN-Tg skeletal muscle were probed with SSPN-specific antibodies (R15) that cross-react with SSPN, as expected (**f**). However, nSPN antibodies (R20) do not cross-react with SSPN (**e**). Table 2 summarizes antibodies used in the study. Molecular weight markers (kDa) are provided on the *left*

In order to assess the expression nSPN at the protein level, we generated polyclonal antibodies against nSPN by immunizing rabbits with a 12-amino-acid synthetic peptide. The peptide sequence was chosen to span the junction between exon 1 and exon 4, which includes six amino acids encoded by each exon (Fig. 1c, double underline). Specificity of nSPN antibodies was determined using multiple assays. First, we demonstrate that affinity-purified R20 antibodies only recognized BSA coupled to nSPN Peptide 20 and do not cross-react with BSA conjugated to non-specific peptide or to BSA alone (Fig. 3a). Second, the specificity of R20 was investigated using peptide competition experiments, as previously described [1, 25]. R20 antibodies recognized nSPN (~11 kDa) in microsomes prepared from rabbit and mouse skeletal muscles (Fig. 3b). We found that R20 binding to nSPN was inhibited in the presence of nSPN Peptide 20 (Fig. 3c), but not in the presence of a non-specific control peptide (Fig. 3d). Lastly, we demonstrate that nSPN antibodies do not cross-react with μSPN by probing protein lysates from wild-type (non-transgenic) and μSPN-transgenic (Tg) skeletal muscle. μSPN-specific antibodies (Rabbit 12 [22]) bind to the μSPN-Tg protein (Fig. 3e). However, nSPN antibodies do not cross-react with μSPN (Fig. 3e). To demonstrate that nSPN antibodies do not cross-react with SSPN, we probed protein lysates from wild-type (non-transgenic) and SSPN-Tg skeletal muscle. SSPN-specific antibodies (Rabbit 15 [21]) bind to the SSPN-Tg protein (Fig. 3f). However, nSPN antibodies do not cross-react with SSPN (Fig. 3f). We used transgenic overexpression models for these experiments due to the abundance of μSPN and SSPN that is amenable to robust protein detection.

### Analysis of membrane fractions reveals that nSPN is enriched in SR and is lost in δ-SG nulls

**Fig. 4:**
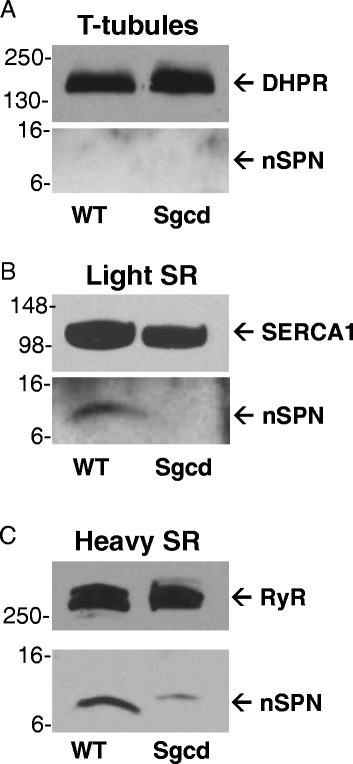
Analysis of nSPN in isolated T-tubule and sarcoplasmic reticulum membranes. T-tubule sarcoplasmic reticulum (*SR*) membranes were prepared from skeletal muscle from control wild-type (*WT*) and δ-SG-null (*Sgcd*) mice. Samples analyzed include T-tubules (**a**), light SR (**b**), and heavy SR (**c**). Immunoblotting of DHPR, SERCA1, and RyR in different fractions of muscle microsomes after sucrose gradient centrifugation to separate T-tubule and SR fractions. Previous studies have shown that γ-SG, δ-SG, δ-SG3, and μSPN are present in the light SR of control mice, but are absent in the light SR of δ-SG-null mice [26]. Additionally, our previous analysis using identical preparations demonstrated δ-SG3 and μSPN are enriched in the heavy SR of wild-type muscle, but are lost in the heavy SR of the δ-SG-null mice. Molecular weight markers (kDa) are provided on the *left*

Affinity-purified antibodies were used to investigate nSPN expression in preparations of skeletal muscle membranes. Specifically, T-tubule, light SR, and heavy SR membranes from skeletal muscle of wild-type mice were isolated by sucrose gradient ultracentrifugation, as previously described [26]. The T-tubule membranes are invaginations of the sarcolemma that contain a large number of L-type calcium channels (DHPR). SR membranes are biochemically segregated into two functional domains, a longitudinal region (light SR) with SERCA1 enrichment and a triadic (heavy) region (heavy SR) composed of terminal cisternae, which are adjacent to each T-tubule and contain proteins such as RyR, triadin, and calsequestrin [36]. Immunoblot analysis of these fractions reveals that nSPN is not present in T-tubule membrane preparations, but is very abundant in heavy SR membranes, while low levels of nSPN are detectable in light SR (Fig. 4a–c). As controls, DHPR, RyR, and SERCA are shown.

It is well established that SSPN interacts with the SGs at the sarcolemma [9, 34, 37] and that μSPN interacts with a variation in the SGs in the SR [25, 26]. In these experiments, both SSPN and μSPN are lost in multiple SG-deficient mouse models [38–40]. We recently demonstrated that loss of SSPN in SSPN-null skeletal and heart muscles significantly decreases abundance of the SGs leading to reduced strength [41, 42]. In order to determine whether nSPN is affected by loss of the SGs, we analyzed purified T-tubule, light SR, and heavy SR fractions from skeletal muscle of Sgcd^−/−^ mice. Loss of δ-SG leads to the complete absence of the other SGs [43], and we did not detect any SG contamination in the preparations (data not shown). Previous studies have shown that γ-SG, δ-SG, δ-SG3, and μSPN are present in the light SR of control mice, but are absent in the light SR of Sgcd^−/−^ mice [26]. Additionally, our previous analysis using identical preparations demonstrated that δ-SG3 and μSPN are enriched in the heavy SR of wild-type muscle, but are lost in the heavy SR of the Sgcd^−/−^ mice. We found that nSPN was diminished or absent from heavy SR and light SR fractions in the Sgcd^−/−^ samples, respectively (Fig. 4a–c). These data suggest that nSPN’s localization to the SR may be dependent on δ-SG, where it has the potential to form a subcomplex with the other SGs and μSPN. It is also feasible that nSPN is lost as a secondary consequence of the disease process, and additional experiments will be needed to distinguish between these possibilities.

### Subcellular localization of nanospan

**Fig. 5:**
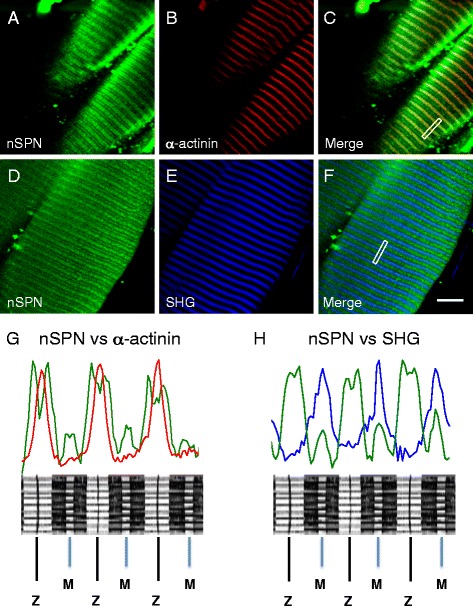
Nanospan localization is consistent with triad localization in mildly stretched skeletal muscle fibers. TPLSM study of nSPN localization in stretched FDB skeletal muscle fibers from wild-type (C57-BL/6J) mice. **a**, **b** TPLSM images of an FDB muscle simultaneously labeled with nSPN (Alexa 488) and α-actinin (Texas Red) antibodies, respectively. **c** The superposition of the images on **a** and **b. d**, **e** Simultaneous TPLSM images of immunolocalization of nSPN and the SHG obtained from an FDB muscle labeled with an antibody against nSPN (Alexa 488), respectively. **f** The superposition of the images on **d** and **e. g** The superposition of the Alexa 488 (nSPN, *green trace*) and Texas Red (α-actinin, *red trace*) fluorescence plot profiles of the fibers shown in **a**–**c**. **h** The superposition of the Alexa 488 emission (nSPN, *green trace*) and SHG (*blue trace*) profiles of the fibers shown in **d**–**f** (profiles taken from areas indicated with *boxes*). The schematic diagrams at the *bottom* of **g** and **h** show the correspondence of the intensity profiles with main sarcomeric hallmarks. Sarcomeric length of the fibers presented in this figure is ~3 μm. *Scale bar*, 10 μm

To more precisely localize nSPN within the SR, we used two-photon laser scanning microscopy (TPLSM) to image wild-type flexor digitorum brevis and interosseous muscles labeled with nSPN antibodies and visualized by indirect immunofluorescence. Three regions of SR membranes have been defined based on evidence from electron microscopy [44–46]: a triadic region flanking the T-tubules (triadic SR), an SR region across the Z-line (ZSR; [45]), and an SR region across the M-line (LSR; [45]). Note that both the ZSR and LSR are represented in light SR fractions, while the triadic SR is represented in the heavy SR fractions in biochemically isolated membranes from skeletal muscle. The precise position of nSPN with respect to sarcomere structures was determined based on its relative distribution against two landmarks of the sarcomere: (i) the A-band centered at the M-lines, indicated by the fluorescence generated by SHG [47], and (ii) the Z-lines, indicated by labeling with α-actinin-specific antibodies. The data in Fig. 5 reveal the location of nSPN in stretched fibers relative to the fluorescence intensity from α-actinin antibodies and SHG fluorescence signal.

As shown in the TPLSM images and highlighted in the intensity profiles of Fig. 5, nSPN is distributed in alternating bands within slightly stretched fibers (~3 μm) from WT mice. One band is relatively thicker and brighter, displaying two peaks, and the alternate band is thinner and fainter, displaying a single peak. As expected, α-actinin (red) is visualized as thin, sharp transversal bands (Fig. 5b). The overlay image in Fig. 5c and the profiles in Fig. 5g demonstrate that the two peaks of the wide band of nSPN are centered on the α-actinin band (i.e., the Z-line, also Additional file 2: Figure S2 A–C and G), while the single-peaked band localizes halfway between two consecutive α-actinin bands. A similar result was obtained in another muscle in which the localization of nSPN (Fig. 5d) is compared with the maxima of SHG images (Fig. 5e). In this case, both the overlay image (Fig. 5f) and the intensity profile (Fig. 5h) demonstrate that there is minimal nSPN expression at the SHG peak (i.e., the M-line) and that the double-peaked band is located midway between two consecutive SHG maxima (i.e., the Z-line). The schematics of the sarcomere in Fig. 5g, h illustrate the position of nSPN with respect to contractile hallmarks. TPLSM images from WT muscles that are slightly more stretched (~3.7 μm) showed a similar nSPN distribution except for the fact that the thinner and fainter band that co-localizes with the SHG maxima is lost in these images (Additional file 2: Figure S2). This last result indicates that the nSPN signal centered at the M-line becomes reduced below the detection level in the more stretched muscle fibers.

**Fig. 6:**
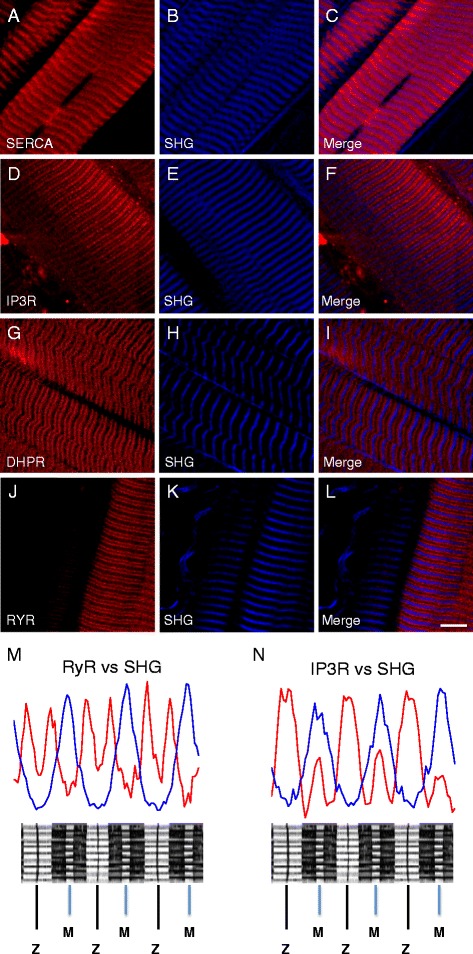
Immunolocalization of muscle proteins using two-photon laser scanning microscopy. Two-photon laser scanning microscopy (TPLSM) was used to study the localization of DHPR, RYR-1, SERCA, and IP3R expression in skeletal muscle fibers from wild-type (C57-BL/6J) mice. **b**, **e**, **h**, **k** TPLSM images of the SHG signals obtained simultaneously to the fluorescent signal from fibers labeled with antibodies against SERCA (**a**, Texas Red), IP3R (**d**, Texas Red), DHPR (**g**, Texas Red), and RyR-1 (**j**, Texas Red). **c**, **f**, **i**, **l** The superposition of the images of the Texas Red fluorescence and SHG signals obtained from the different muscles. **m** The superposition of the Texas Red and SHG fluorescence plot profiles of the fiber shown in **j**–**l** and the relative localization of the RyR-1 expression in the sarcomere. **n** The superposition of the Texas Red and SHG fluorescence plot profiles of the fiber shown in **d**–**f** and the relative localization of the IP3R expression in the sarcomere. *Scale bar*, 10 μm

The fluorescence patterns observed in the muscle fibers with nSPN labeling resemble those observed with other SR transmembrane proteins such as SERCA and IP3R (Fig. 6a–c and Fig. 6d–f, respectively). SERCA and IP3R have been previously shown by immunohistochemistry to be expressed in both the SR centered at the Z-line (ZSR) and the SR centered at the M-line (LSR) [48]. On the other hand, immunolocalization of proteins expressed only at the triadic junctions such as αDHPR and RyR1 always displays a double-banding pattern flanking the Z-line (Fig. 6g–i and Fig. 6j–l, respectively). In these images, note the shorter distance across the Z-line than that across the M-line. Taken together, results from biochemical isolation of skeletal muscle membranes and TPLSM demonstrate that nSPN is expressed in the SR. Moreover, TPLSM reveals that nSPN is differentially expressed in the SR membrane, being more highly expressed in the SR centered at the Z-line (both in the ZSR and triadic SR) and less highly expressed in the SR centered at the M-line (thinner and fainter bands in the immunofluorescence images).

**Fig. 7:**
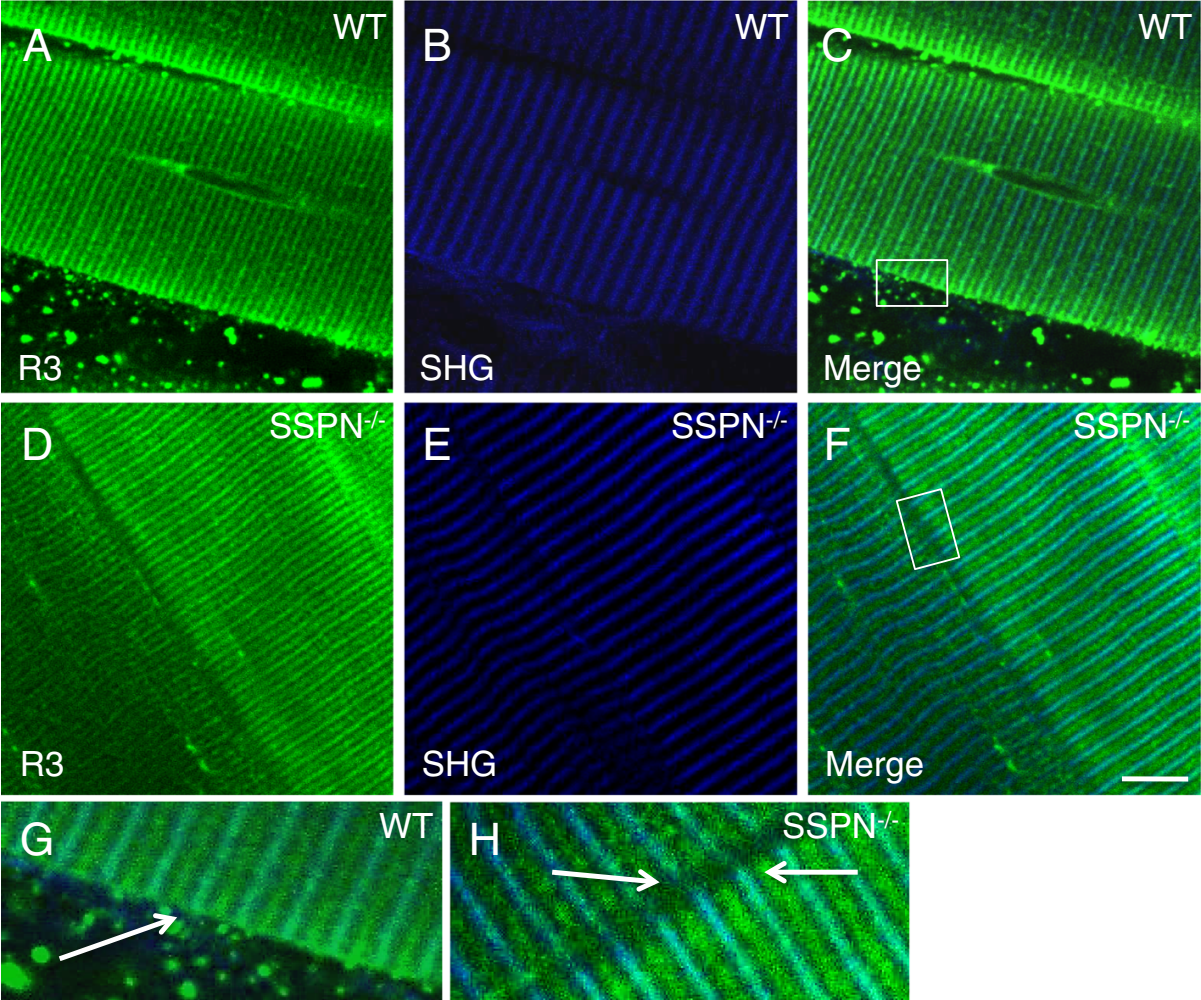
Full-length SSPN is expressed only in the surface membrane and loss of SSPN does not affect nSPN localization. **a**–**c** Simultaneous TPLSM images of immunolocalization of SSPN, μSPN, nSPN (**a**) and SHG (**b**) obtained from an FDB muscle isolated from wild-type mice and labeled with affinity-purified Rabbit 3 (R3) polyclonal antibodies that recognize an N-terminal region that is common to all three SSPN isoforms (Alexa 488). **c** The superposition of the images on **a** and **b**. **d**–**f** Simultaneous TPLSM images of immunolocalization of nSPN (**d**) and SHG (**e**) obtained from an FDB muscle isolated from SSPN-null mice and labeled with Rabbit 3 (R3) polyclonal antibodies (Alexa 488). Note that exon 2 and part of exon 3 were targeted for deletion in the SSPN nulls [28], which disrupts SSPN and μSPN, but not nSPN, expression. **f** The superposition of the images on **d** and **e**. **g** A magnified view of the area enclosed by the *white rectangle* in **c**. **h** A magnified view of the area enclosed by the *white rectangle* in **f**. *White arrows* in **g** and **h** point to the presence (**g**) or absence (**h**) of sarcolemmal labeling in the wild-type and SSPN-null mice, respectively. Sarcomere length ~3 μm. *Scale bar*, 10 μm

We next addressed the question of whether nSPN can be detected in fibers from SSPN-null mice, which were generated by targeted knockout of exon 2, intron 2, and part of exon 3 [28]. In co-localization experiments, we found that nSPN is expressed in SSPN-null mice and that the distribution pattern of nSPN is similar to the pattern that we documented in wild-type muscle at similar sarcomere lengths (compare Fig. 7a–c with Fig. 7d–f). Moreover, when we compared detailed staining patterns of muscle fibers from wild-type and SSPN-null muscles labeled with SSPN antibodies, we were able to distinguish between cell surface expression and intracellular SR membrane expression. Note that SSPN antibodies were generated to a peptide located within exon 1, which is common to all three isoforms of SSPN. Exon 2 and part of exon 3 were targeted for deletion in the SSPN-deficient mice [28], which is predicted to disrupt SSPN and μSPN, but not nSPN expression. SSPN was not detected at the sarcolemma of SSPN-deficient fibers (Fig. 7h), as expected and shown previously [28]. Immunoblot analysis of protein lysates using nSPN-specific antibodies revealed increased abundance of nSPN in SSPN-deficient muscle (Additional file 3: Figure S3). This raises the possibility that nSPN may compensate for SSPN within the DGC of SSPN-deficient muscle. To investigate this possibility, we purified the DGC from SSPN-deficient muscle with the rationale that interaction with the DGC is required in order to compensate for SSPN. However, nSPN did not co-purify with the DGC complex in either wild-type or SSPN-deficient muscles (Additional file 3: Figure S3). These results reveal that overall nSPN protein abundance is increased and localized to the sarcolemma in a heterogeneous fashion in the context of SSPN deficiency.

Exon 3, encoding transmembrane domains 3 and 4 in SSPN, is absent in nSPN and μSPN. Through immunofluorescence analysis, we have observed that SSPN is the only isoform to localize to the sarcolemmal membrane in wild-type muscle, while nSPN and μSPN localize to internal SR membranes. These data suggest that protein regions encoded by exon 3, which include transmembrane domains 3 and 4, the large extracellular loop, and the intracellular C-terminus, are responsible for cell surface localization. In support of this, our prior analysis of deletion mutants identified regions in the large extracellular loop that interact with the intact, tetrameric (α-, β-, γ-, δ-SG) subcomplex [9, 34, 37].

### Nanospan is enriched in type I muscle fibers and reduced in DMD

**Fig. 8:**
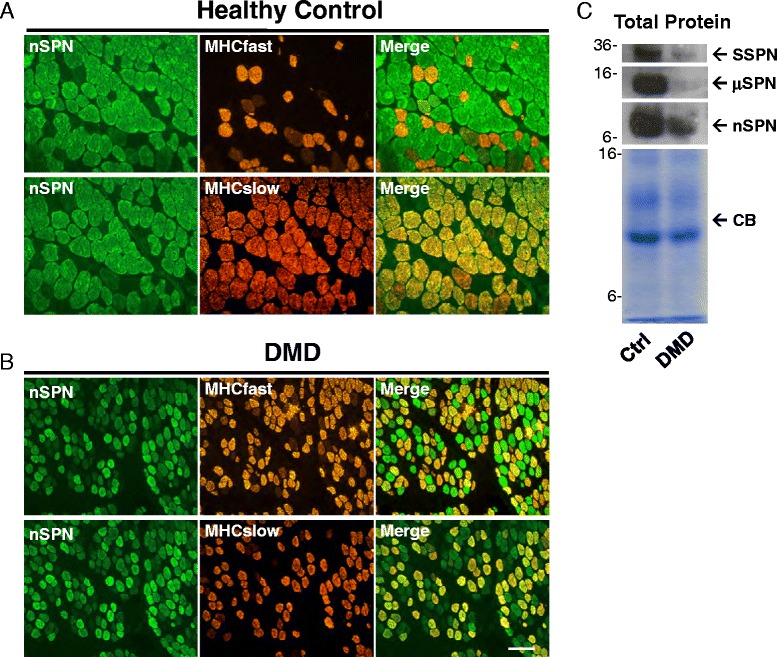
nSPN exhibits fiber type-specific expression in human skeletal muscle. **a**, **b** Localization of nSPN in skeletal muscle biopsies taken from control human (**a**) and DMD patients (**b**). Transverse muscle cryosections (8 μm) were stained with affinity-purified antibodies to nSPN (Rabbit 20) and detected using FITC (*green*). In order to determine whether nSPN is preferentially expressed in either fast- or slow-twitch muscle, sections were co-stained with MHCfast and MHCslow, as indicated. nSPN is preferentially expressed in intracellular membrane compartments within slow-twitch muscle. Merged images are shown in *right panels. Scale bar*, 20 μm. **c** Skeletal muscle lysates from normal control and DMD biopsies were analyzed by SDS-polyacrylamide gel electrophoresis and immunoblotted using antibodies recognizing SSPN, μSPN, and nSPN. Protein expression levels of SSPN and μSPN are dramatically reduced in DMD muscle lysates. nSPN is only moderately reduced in DMD samples. Coomassie blue (*CB*)-stained protein gels are shown as loading controls. Molecular weight markers (kDa) are provided on the *left*

Several forms of muscular dystrophy are caused by genetic mutations in genes encoding protein components of the DGC. Duchenne muscular dystrophy (DMD) is caused by mutations in the dystrophin gene resulting in progressive muscle wasting and weakness [49]. Dystrophin and the other DGC components are lost from the sarcolemma in muscle from DMD patients. Using affinity-purified nSPN antibodies, we examined nSPN expression in muscle from DMD patients by immunoblot analysis and indirect immunofluorescence. We examined several DMD patients with primary mutations in dystrophin and no detectable dystrophin protein. Indirect immunofluorescence assays with antibodies against the C-terminus of dystrophin verify dystrophin is completely absent from the DMD sarcolemma (data not shown). Without dystrophin, the DGC is reduced, likely the result of rapid protein degradation. Skeletal muscle cryosections from normal and DMD patients were examined for nSPN expression by indirect immunofluorescence. In transverse sections, we observed an intracellular staining pattern for nSPN in some, but not all, fibers in normal human skeletal muscle biopsies (Fig. 8a), suggesting fiber type-specific expression. In co-staining experiments with slow (type I) and fast (type II) myosin heavy chain (MHC) antibodies, we found that nSPN expression is limited to slow-twitch muscle fibers in normal healthy controls (Fig. 8a). Similarly, nSPN is robustly expressed in slow fibers from DMD patients (Fig. 8b). Immunoblot analysis reveals a dramatic decrease in nSPN levels in DMD protein lysates relative to controls. This may be due to the decreased abundance of slow fibers in the DMD samples (Fig. 8c). We now show that μSPN protein is dramatically decreased in DMD muscle protein lysate samples using immunoblot analysis (Fig. 8c). Similarly, nSPN expression was reduced in DMD muscle lysates when compared to normal controls (Fig. 8c). The blots of μSPN and nSPN in control and DMD are from identical exposures with an antibody that cross-reacts with both proteins (Fig. 8c). These data suggest that nSPN and μSPN are in relatively equal quantities in control human skeletal muscle.

## Discussion

**Fig. 9:**
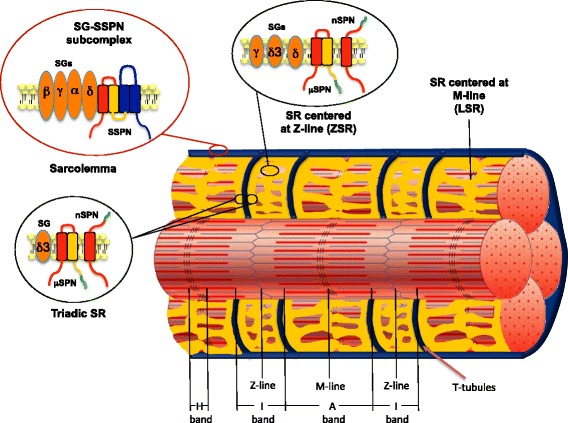
Alternative splicing produces SSPN, μSPN, and nSPN with distinct subcellular membrane localizations. The schematic diagram illustrates the sarcolemma, SR, and T-tubule membranes and their placement with respect to the muscle sarcomere. The SR has three distinct regions: a triadic region flanking the T-tubules (triadic SR), a region across the Z-line (ZSR), and a region across the M-line (LSR). Note that the ZSR and LSR are represented in light SR fractions, while the triadic SR is represented in the heavy SR fractions. The *upper left enlargement* represents a schematic diagram showing localization of SSPN at the sarcolemma, where it is a core component of the DGC. SSPN is represented as a four-transmembrane-spanning protein with the color scheme as described in Fig. 1. The sarcoglycans (α-, β-, γ-, δ-SGs; *orange*) are depicted as transmembrane *oval shapes*. The SGs and SSPN form a tight subcomplex within the DGC. Dystrophin (not shown) interacts with β-DG on the cytoplasmic face of the sarcolemma membrane and provides linkage to the actin cytoskeleton. Dystroglycan (not shown) serves as a receptor for extracellular matrix ligands. The *enlargement on the upper right* represents a schematic illustration of the SR membranes where nSPN and μSPN (color scheme as described in Fig. 1) are enriched and associate with a subset of the SGs (δ-SG, δ-SG3, and γ-SG; *orange*) [26, 50]. Triadic SR regions contain δ-SG3, nSPN, and μSPN (*lower enlargement*), but lack δ-SG and γ-SG as previously described [26, 50]. Data from two-photon laser scanning microscopy reveals that nSPN is mostly associated with the ZSR and the triadic SR, and only minimally with the LSR. While protein expression of nSPN, μSPN, and SG isoforms in the SR is demonstrated, it is unclear whether these proteins form a subcomplex within these intracellular membrane structures, which will be the focus of future studies

In the current study, we report the isolation of nSPN, an alternatively spliced isoform of SSPN. nSPN possesses only one TM domain and is localized to the SR. Moreover, nSPN expression is enriched in the region of the SR centered at the Z-line, the ZSR, and the triadic SR, while minimally present in the LSR region of the SR centered at the M-line (Fig. 9). In fact, nSPN is not retained by sWGA chromatography, the first step in DGC purification, suggesting that it does not associate with proteins containing O-GlcNAc modification. Thus, nSPN is separated from the DGC during an early fractionation step. Taken together, these data demonstrate nSPN is not an integral component of the DGC that is present at the sarcolemma.

We have identified two proteins produced from alternatively splicing of SSPN mRNA designated as microspan (μSPN) [25] and nanospan (nSPN) (this paper). SSPN, μSPN, and nSPN each have the same first transmembrane domain that is encoded by exon 1. μSPN and nSPN lack transmembrane domains 3 and 4 as well as the large extracellular loop (LEL) that is specific to SSPN. Neither μSPN nor nSPN is associated with the DGC at the sarcolemma, suggesting that their functions are entirely distinct from those of SSPN, suggesting that the domains unique to SSPN (i.e., TM3, TM4, and the LEL) may contain the sarcolemma-targeting domain(s) (Fig. 9). μSPN is localized to the SR, and we have shown overexpression of μSPN causes severe disruption of triad development in skeletal muscle of transgenic mice [25].

Several components of the DGC have been localized to intracellular membrane structures in skeletal muscle. Ueda and colleagues have reported the expression of both δ- and γ-SG to the SR in addition to their well-established localization to the sarcolemma [23]. Interestingly, a splice variant of δ-SG (δ-SG3) localizes to the SR was identified by the research group of Coral-Vázquez [26]. δ-SG3 lacks 122 C-terminal amino acids of full-length δ-SG, including the conserved Cys-rich extracellular domain. The C-terminus of δ-SG3 consists of 10 amino acids, which are encoded by a newly identified exon 5b [26, 50]. While we demonstrate protein expression of nSPN, μSPN, and SG isoforms in the SR, it is unclear whether these proteins form a subcomplex within these intracellular membrane structures. Given the tight biochemical association of the SG-SSPN complex at the sarcolemma, it is feasible that a variation in the SG-SSPN subcomplex is present in the SR of striated muscle. Furthermore, the identification of several splice variants that localize exclusively to the SR suggests that these proteins may function in Ca^2+^ regulation (Fig. 9). This hypothesis is further strengthened by the observation that both the murine and hamster models of δ-SG deficiency display aberrant intracellular Ca^2+^ handling in skeletal muscle [26, 50, 51]. Future studies will focus on determining whether the SG-SSPN isoforms that are localized to the SR truly form a subcomplex and whether perturbations in the complex affect intracellular Ca^2+^ levels.

## Conclusions

- In the present work, we identify nSPN as an alternatively spliced isoform of SSPN that is generated from the splicing of exon 1 to exon 4. Exon 4 encodes six amino acids. nSPN is predicted to contain only one transmembrane domain, so that the N- and C-terminal regions are on the luminal and cytosolic faces of the membrane, respectively.
- Analysis of gene expression reveals that nSPN is enriched in skeletal and cardiac muscles.
- Using multiple approaches including biochemical fractionation and TPLSM, we demonstrate that nSPN is localized to specific structures at the SR membranes, particularly enriched at the ZSR and the triadic SR, and much less highly expressed at the LSR regions centered at the M-line, as illustrated in Fig. 9.
- Loss of SSPN and μSPN by targeted deletion of exon 2, intron 2, and exon 3 does not affect localization of nSPN to the SR, but SSPN deficiency does lead to increased abundance of nSPN that does not associate with the DGC.
- nSPN expression is lost in muscle from Sgcd^−/−^ mice, a model for limb girdle muscular dystrophy type 2F.
- In human muscle biopsies, nSPN expression is restricted to slow muscle fibers. nSPN expression is reduced, but not absent, in skeletal muscle from Duchenne muscular dystrophy cases.
- In summary, alternative splicing of SSPN mRNA generates three protein isoforms (SSPN, μSPN, and nSPN) that differ in the number of transmembrane domains affecting subcellular membrane association into distinct protein complexes.

## Additional files

Additional file 1: Figure S1. nSPN mRNA transcripts are predominant in skeletal and heart muscles. RT-PCR was performed on cDNA isolated from various human tissues (skeletal muscle, testis, colon, heart, brain, breast, and bone marrow) using a forward primer in exon 1 and a reverse primer in exon 4, as illustrated in Fig. 1c. PCR products were obtained for μSPN (179 bp) and nSPN (90 bp). RT-PCR performed without template DNA is shown as a negative control (neg. ctrl). Molecular size markers are indicated on the left. (PDF 348 kb)

Additional file 2: Figure S2. Nanospan localization is consistent with triad localization in stretched skeletal muscle fibers. TPLSM study of nSPN localization in stretched FDB skeletal muscle fibers from wild-type (C57-BL/6J) mice. Panels A and B are TPLSM images of an FDB muscle simultaneously labeled with nSPN (Alexa 488) and α-actinin (Texas Red) antibodies. Panel C shows the superposition of the images on panels A and B. Panels D and E show simultaneous TPLSM images of immunolocalization of nSPN and the SHG obtained from an FDB muscle labeled with an antibody against nSPN (Alexa 488). Panel F shows the superposition of the images on panels D and E. Panel G corresponds to the superposition of the Alexa 488 (nSPN, green trace) and Texas Red (α-actinin, red trace) fluorescence plot profiles of the fibers showed in panels A–C. Panel H corresponds to the superposition of the Alexa 488 emission (nSPN, green trace) and SHG (blue trace) profiles of the fibers shown in panels D–F (profiles taken from areas indicated with boxes). The schematic diagrams at the bottom of panels G and H show the correspondence of the intensity profiles with main sarcomeric hallmarks. Sarcomeric length of the fibers presented in this figure is ~3.7 μm. Scale bar, 10 μm. (PDF 2028 kb)

Additional file 3: Figure S3. nSPN does not associate with the DGC. Skeletal microsomes from wild-type (WT) and SSPN-deficient muscle was solubilized with digitonin and subjected to sWGA enrichment, which represents the first step in DGC purification. Immunoblot analysis of the starting material (start), sWGA void, and sWGA eluate probed with antibodies to α-DG (as a marker for DGC purification) and nSPN (R20). (PDF 118 kb)

## Abbreviations

AR-LGMD: Autosomal recessive limb girdle muscular dystrophy
DG: Dystroglycan
DGC: Dystrophin-glycoprotein complex
DMD: Duchenne muscular dystrophy
nSPN: Nanospan
SG: Sarcoglycan
SHG: Second harmonic generation
SR: Sarcoplasmic reticulum
SSPN: Sarcospan
TPLSM: Two-photon laser scanning microscopy
μSPN: Microspan
