# Supplementary figures and images for: Nanospan, an alternatively spliced isoform of sarcospan, localizes to the sarcoplasmic reticulum in skeletal muscle and is absent in limb girdle muscular dystrophy 2F
Source: Skelet Muscle. 2017 Jun 6;7:11. doi: 10.1186/s13395-017-0127-9 (PMC5461684; doi:10.1186/s13395-017-0127-9)

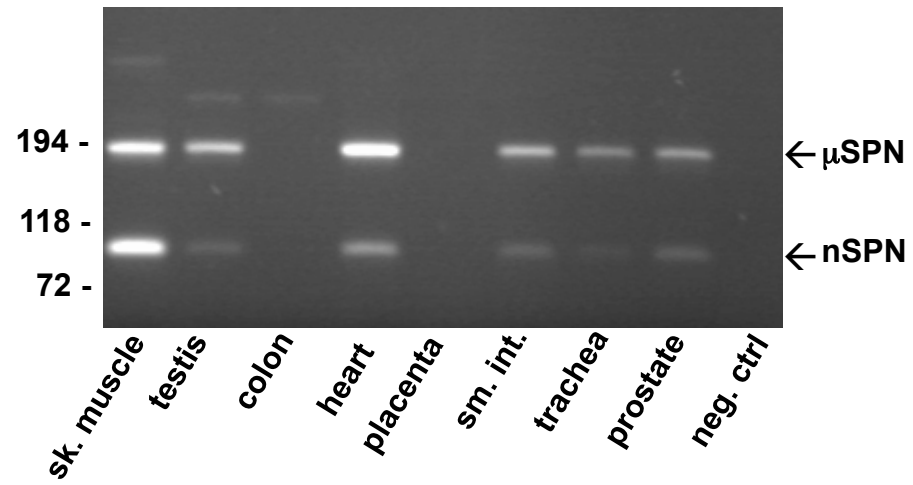

Figure S1

Supplement: Supplementary file 1 — nSPN mRNA transcripts are predominant in skeletal and heart muscles. RT-PCR was performed on cDNA isolated from various human tissues (skeletal muscle, testis, colon, heart, brain, breast, and bone marrow) using a forward primer in exon 1 and a reverse primer in exon 4, as illustrated in Fig. 1c. PCR products were obtained for μSPN (179 bp) and nSPN (90 bp). RT-PCR performed without template DNA is shown as a negative control (neg. ctrl). Molecular size markers are indicated on the left. (PDF 348 kb) [file 13395_2017_127_MOESM1_ESM.pdf]

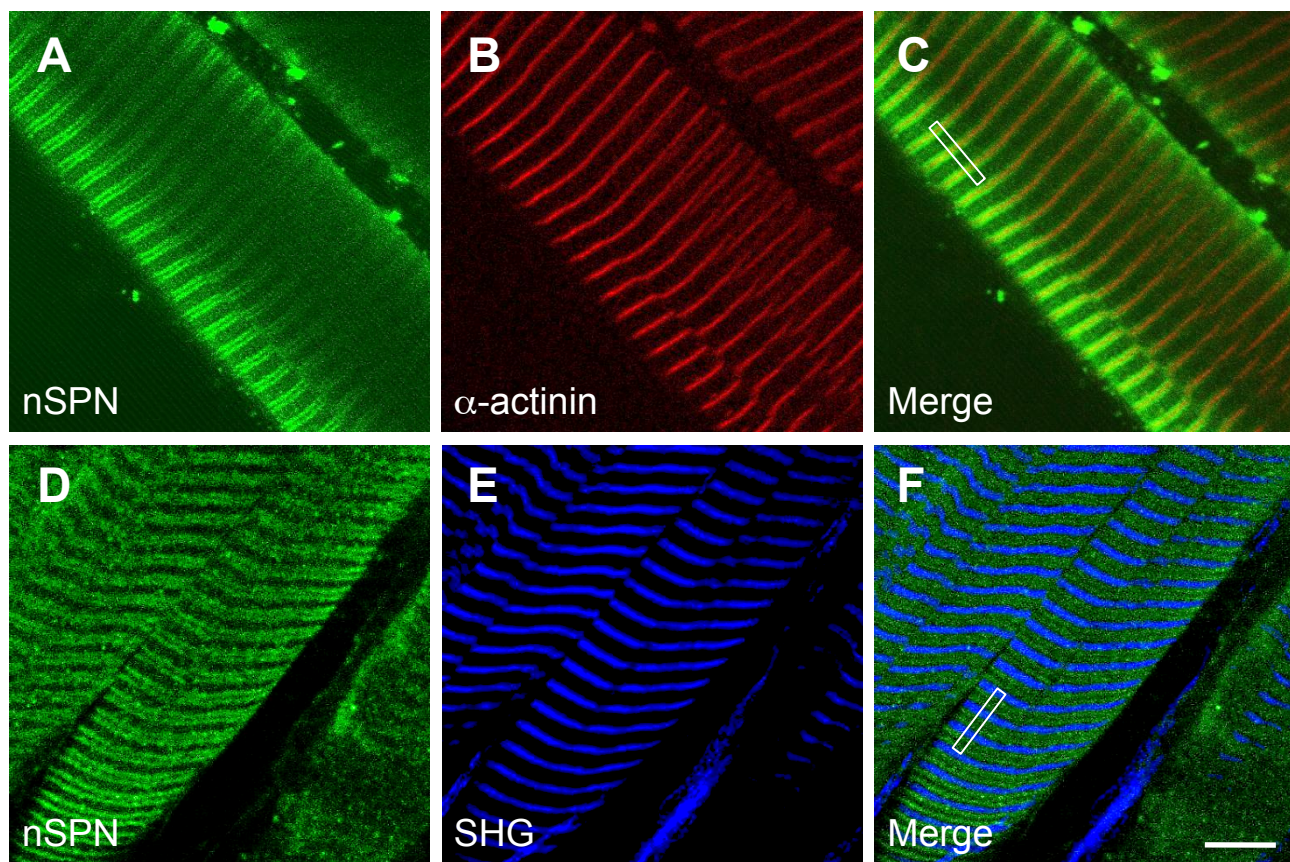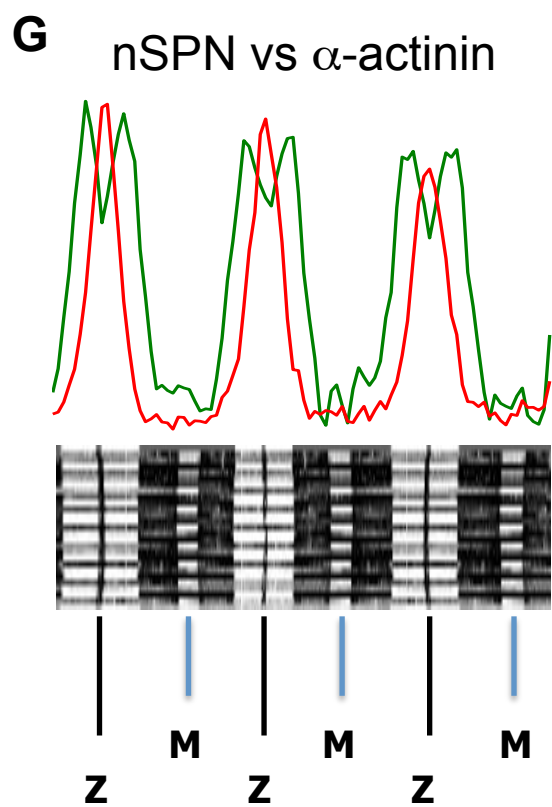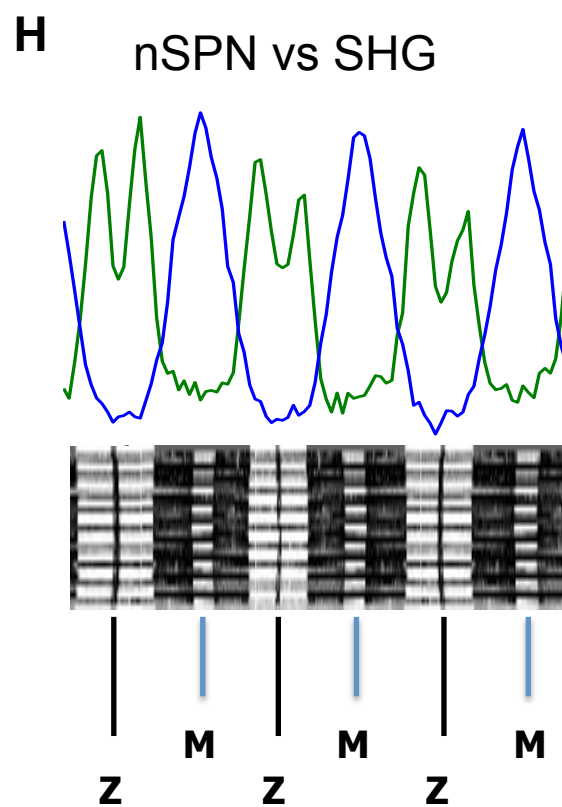

Supplement: Supplementary file 2 — Nanospan localization is consistent with triad localization in stretched skeletal muscle fibers. TPLSM study of nSPN localization in stretched FDB skeletal muscle fibers from wild-type (C57-BL/6J) mice. Panels A and B are TPLSM images of an FDB muscle simultaneously labeled with nSPN (Alexa 488) and α-actinin (Texas Red) antibodies. Panel C shows the superposition of the images on panels A and B. Panels D and E show simultaneous TPLSM images of immunolocalization of nSPN and the SHG obtained from an FDB muscle labeled with an antibody against nSPN (Alexa 488). Panel F shows the superposition of the images on panels D and E. Panel G corresponds to the superposition of the Alexa 488 (nSPN, green trace) and Texas Red (α-actinin, red trace) fluorescence plot profiles of the fibers showed in panels A–C. Panel H corresponds to the superposition of the Alexa 488 emission (nSPN, green trace) and SHG (blue trace) profiles of the fibers shown in panels D–F (profiles taken from areas indicated with boxes). The schematic diagrams at the bottom of panels G and H show the correspondence of the intensity profiles with main sarcomeric hallmarks. Sarcomeric length of the fibers presented in this figure is ~3.7 μm. Scale bar, 10 μm. (PDF 2028 kb) [file 13395_2017_127_MOESM2_ESM.pdf]

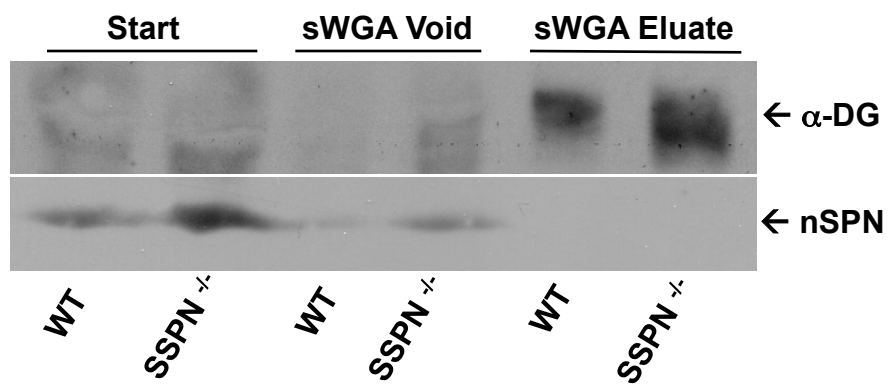

Supplement: Supplementary file 3 — nSPN does not associate with the DGC. Skeletal microsomes from wild-type (WT) and SSPN-deficient muscle was solubilized with digitonin and subjected to sWGA enrichment, which represents the first step in DGC purification. Immunoblot analysis of the starting material (start), sWGA void, and sWGA eluate probed with antibodies to α-DG (as a marker for DGC purification) and nSPN (R20). (PDF 118 kb) [file 13395_2017_127_MOESM3_ESM.pdf]
